# Supplementary material for: Determinants of blood pressure and blood glucose control in patients with co-morbid hypertension and type 2 diabetes mellitus in Ghana: A hospital-based cross-sectional study
Source: PLOS Glob Public Health. 2022 Dec 6;2(12):e0001342. doi: 10.1371/journal.pgph.0001342 (PMC10022155; doi:10.1371/journal.pgph.0001342)
Supplement: Attachment — Submitted filename: Response to reviewers.docx [file pgph.0001342.s001.docx]

The Editor in Chief

Plos Global Public Health

Dear Sir,

**Re: Determinants of blood pressure and blood glucose control in patients with co-morbid hypertension and type 2 diabetes mellitus in Ghana: A hospital-based cross-sectional study.**

I wish to thank the editor and reviewers for their time, comments, and expertise advice on the above title. Please find below Author’s response.

Yours faithfully,

Adwoa Oforiwaa Kwakye (Ph.D.)

**Determinants of blood pressure and blood glucose control in patients with co-morbid hypertension and type 2 diabetes mellitus in Ghana: A hospital-based cross-sectional study.**

| **Section** | **Second Reviewer’s comments** | **Author’s response** |
| --- | --- | --- |
| Study design and context | Please clarify if the facility is a public or a private health facility. | Rectified. It is a public health facility. |
|  | Also provide some information regarding whether Ghana has an NCD control  program in place or not. Please mention the services that are available – e.g., identification, management, follow-up services, monitoring of clinical status, etc - and whether services such as testing, and provision of medicines are available to patients free of cost or not. | Rectified. The information has been provided. |
| Results | Table 4 has some contradictory findings between the "controlled BP group"  and "controlled blood glucose group" (eg. patients who take 2 or 3 daily doses of medication had better odds of BP control compared to single daily dose, while the opposite was true for blood glucose control). Can these differences be explained? | Further investigation revealed that among participants, the uptake of two or three daily dose of medication was highly prevalent among participants who had controlled BP, but uncontrolled glucose (78.1%, n=50/64) compared to those with uncontrolled BP and uncontrolled glucose (55.8%, n=43/77), uncontrolled BP but controlled glucose (41.4%, n=48/116), and those with controlled BP and controlled glucose (59.7%, n=43/72). This shows that those with only uncontrolled glucose levels were more likely to be on medication that is taken 2 or 3 times daily compared to any other group.  The explanation has been provided. Thanks |
| Discussion | please provide some information about the status of the NCD program in Ghana  and in the health facility in particular. In particular, are there any implementation challenges in the program which may affect the treatment services and ultimately affect the control of BP and blood glucose among patients? For example, if availability of medications is frequently disrupted, patients may have to go to private health facilities or pharmacies to buy their medication, which would lead to higher out of pocket expenditure and affect their control of BP and blood glucose. Are all the commonly prescribed antihypertensives and oral hypoglycaemic agents included in the national essential drugs list? If not, that would again lead to high out of pocket expenditure. Does the health facility run out of blood sugar testing kits. If yes, that would adversely affect testing services. | Thank you for your comments. Information on the NCD program in Ghana and that of the health facility has been provided. |
| Limitation | In the Limitations section, do mention that since the study focused on the patients who were already diagnosed and seeking treatment at the facility, this may not reflect the actual proportion of controlled BP and blood glucose in the community, since it is quite likely that there is a considerable number of patients with hypertension and diabetes, who are not yet diagnosed. | This has been rectified and the statement added to the limitation section. |
| Results on adherence | Also, since the study is focused on the control of BP and blood glucose, it would have been ideal if the adherence to treatment was also probed. | Information on adherence to medication has been included in the analysis, results and discussions. |
| Conclusion | I would suggest adding some more recommendations for policymakers -  the factors which are associated with decreased control of BP/blood sugar could be useful in identifying vulnerable groups, and their adherence to medication and hence level of control could potentially be improved by targeted IEC campaigns in the community-by-community health workers. Also, better coordination between levels of care (such as between facility level health workers and community health workers) could result in better patient outcomes (by ensuring improved adherence to treatment, improved health seeking behaviour, etc). | This has been rectified. Thanks |

| **Section** | **First Reviewer’s comment** | **Author’s response** |
| --- | --- | --- |
| Results | slight labelling error in table 4 | Table 4 has been relabeled to *“****Multivariable binary logistic regression model of factors associated with controlled levels of blood pressure, glucose and both blood pressure and glucose among hypertensive and type 2 diabetes mellitus co-morbid patients.”*** |
